# Supplementary material for: HemN2 Regulates the Virulence of Pseudomonas donghuensis HYS through 7-Hydroxytropolone Synthesis and Oxidative Stress
Source: Biology (Basel). 2024 May 24;13(6):373. doi: 10.3390/biology13060373 (PMC11200716; doi:10.3390/biology13060373)
Supplement: Supplementary file 1 [file biology-13-00373-s001.zip › biology-3009666-supplementary.pdf]

Supplementary Materials

Table S1. Strains and plasmids used in this study.

Strains and Plasmids	Description	Source
S17-1(λpir)	*thi pro hsdR recA*; chromosomal RP4-2; (Tc::Mu) (Km::Tn7) Tp^r Sp^r	[38]
OP50	Uracil trophic deficiency type	This study
Pseudomonas donghuensis strains		
HYS	Wild type, lethal to *C. elegans*, Cm^r	This study
HYS1	Δ*hemN1*	This study
HYS2	Δ*hemN2*	This study
HYS3	Δ*hemN3*	This study
HYS4	Δ*hemN4*	This study
HYS5	Δ*hemN1*Δ*hemN2*	This study
HYS6	Δ*hemN1*Δ*hemN2*Δ*hemN4*	This study
HYS7	Δ*hemN2*/pBBR2-*hemN2*	This study
HYS8	Δ*gacS*	[12]
HYS9	Δ*cbrA*	[7]
HYS10	Δ*panB*	[10]
HYS11	Δ*pvdA*	[11]
Plasmids		
pEX18Gm	Gene replacement vector, Gm^r *oriT*$^+$ *sacB*$^+$	[36]
pEX18Gm-*hemN1*-UD	Gene replacement vector for *hemN1*	This study
pEX18Gm-*hemN2*-UD	Gene replacement vector for *hemN2*	This study
pEX18Gm-*hemN3*-UD	Gene replacement vector for *hemN3*	This study
pEX18Gm-*hemN4*-UD	Gene replacement vector for *hemN4*	This study
pBBR1MCS-2	Mobilizable broad-host-range cloning vector, Km^r	[37]
pBBR2-*hemN2*	Cloning vector for *hemN4*	This study

Cm, chloramphenicol, Gm, gentamicin, Km, kanamycin.

Table S2. Oligonucleotide primers used in this study.

Primer	Sequence (5′-3′)[a]	Description
rpoB-RT-1	CGGGAGCGACCAAAGATCAG	Real-time qPCR
rpoB-RT-2	CGTACTCCAGGGCAGCATTG	Real-time qPCR
hemN1-RT-1	GTTGATGGATTGCCTGCACC	Real-time qPCR
hemN1-RT-2	TCAGGCGATTAAAGCCCAGG	Real-time qPCR

hemN2-RT-1	TTCAACCAGGTCAGCATCGG	Real-time qPCR
hemN2-RT-2	TCGATCAGCGAGTGAATCGG	Real-time qPCR
hemN3-RT-1	TCCACAACACCATCGAGCAA	Real-time qPCR
hemN3-RT-2	GCAATCGCCAGTTCGTCATC	Real-time qPCR
hemN4-RT-1	CCCGTACTGCGACTTCAACT	Real-time qPCR
hemN4-RT-2	CGCCGCCAAAGAAGATTGAG	Real-time qPCR
hemN1-up-1	CGGGATCCCGGCGAGAAATAC	Real-time qPCR
hemN1-up-2	GGAATTCCGATCACGGCATAGGT	Real-time qPCR
hemN1-down-1	CGGAATTCCTGACCTGCGGCTT	*Construction of ΔhemN1*
hemN1-down-2	GCTCTAGACCTTGCACCATTGCTCGA	*Construction of ΔhemN1*
hemN1-M-1	GCCCGTTGCCCGTAAA	*Verification of ΔhemN1*
hemN1-M-2	GTCTTGATTACGTCCACCACC	*Verification of ΔhemN1*
hemN2-up-1	CGGGATCCCGGCGAGAAATAC	*Construction of ΔhemN2*
hemN2-up-2	GGAATTCCGATCACGGCATAGGT	*Construction of ΔhemN2*
hemN2-down-1	CGGAATTCCTGACCTGCGGCTT	*Construction of ΔhemN2*
hemN2-down-2	GCTCTAGACCTTGCACCATTGCTCGA	*Construction of ΔhemN2*
hemN2-M-1	GCCCGTTGCCCGTAAA	*Verification of ΔhemN2*
hemN2-M-2	GTCTTGATTACGTCCACCACC	*Verification of ΔhemN2*
hemN3-up-1	CGGAATTCGGGGCGCATCCTCAGTT	*Construction of ΔhemN3*
hemN3-up-2	AGTGTCCTGGTAGGTGTTGAGCGGTCGGGTAGGAGGTGT	*Construction of ΔhemN3*
hemN3-down-1	TCAACACCTACCAGGACACTTTGTCCACCGCTCAACTGGCC	*Construction of ΔhemN3*
hemN3-down-2	CCCAAGCTTGGCGGAACAGAAACTCG	*Construction of ΔhemN3*
hemN3-M-1	CGCCATCTACATCTTCTTCTG	*Verification of ΔhemN3*
hemN3-M-2	TCATCACCCGCATCAACT	*Verification of ΔhemN3*
hemN4-up-1	CGGGATCCAGAACCTGCGGGTGTAT	*Construction of ΔhemN4*
hemN4-up-2	GCTCTAGAGCTGCCGGTGGATGAATT	*Construction of ΔhemN4*
hemN4-down-1	GCTCTAGAGTGGGAGTTGAAGTCGCAGTAC	*Construction of ΔhemN4*

hemN4-down-2	CCCAAGCTTATCCTCAAGGCACGCAAT	*Construction of ΔhemN4*
hemN4-M-1	CGGAGCAGGACTTCATCG	*Verification of ΔhemN4*
hemN4-M-2	GAAACCCGTACCTTCACCAT	*Verification of ΔhemN4*
hemN1/hemN2-up-1	GCTCTAGAAGCACATCCGCAAACAG	*Construction of ΔhemN1ΔhemN2*
hemN1/hemN2-up-2	GGAATTCCAGGCAGTGCAGATAGCG	*Construction of ΔhemN1ΔhemN2*
hemN1/hemN2-down-1	GGAATTCTGGCATCCGCTTCAACG	*Construction of ΔhemN1ΔhemN2*
hemN1/hemN2-down-2	CGGGATCCTCTCGACGACTTCTCCTTCT	*Construction of ΔhemN1ΔhemN2*
hemN1/hemN2-M-1	CTGCGGGAAGATGAACC	*Verification of ΔhemN1ΔhemN2*
hemN1/hemN2-M-2	CCAGTACGACCTGCACAAC	*Verification of ΔhemN1ΔhemN2*
hemN2-1	CCCAAGCTTTTCCGCAGCCTGTCTCG	*Construction of ΔhemN2*/pBBR2-*hemN2*
hemN2-2	CGGGATCCGGACGGTCGTACTTTTGCA	*Construction of ΔhemN2*/pBBR2-*hemN2*
M13-47	CGCCAGGGTTTTCCCAGTCACGAC	PCR universal primer
M13-48	AGCGGATAACAATTTCACACAGGA	PCR universal primer

[a]Restriction sites are underlined.

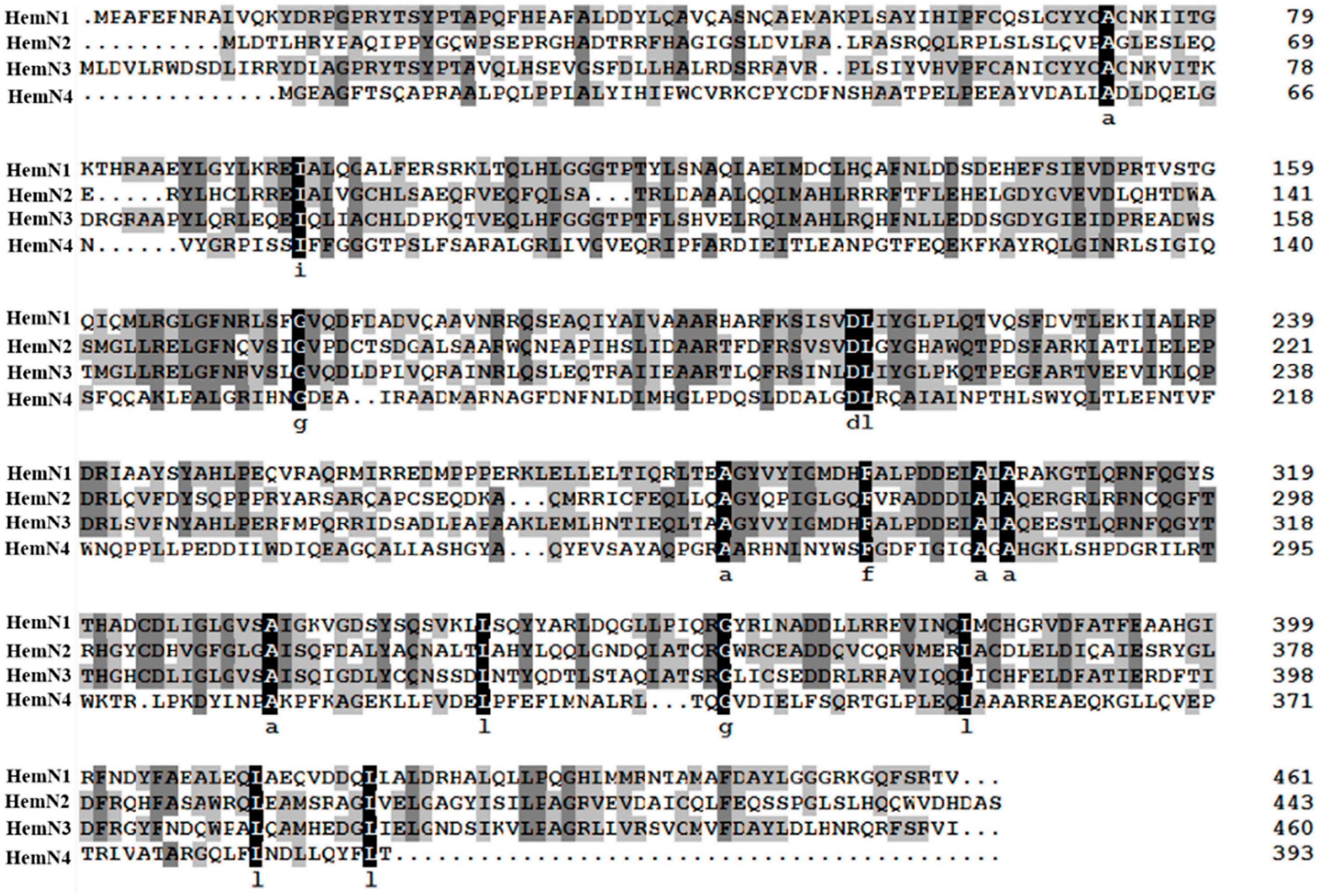

Figure S1. Sequence alignment of HemN proteins of *P. donghuensis* HYS and highlight of the selected residues. Protein sequences for alignment are as follows: HemN1 (WP_010221905.1), HemN2 (WP_050990001.1), HemN3 (WP_010226002.1) and HemN4 (WP_010222203.1).

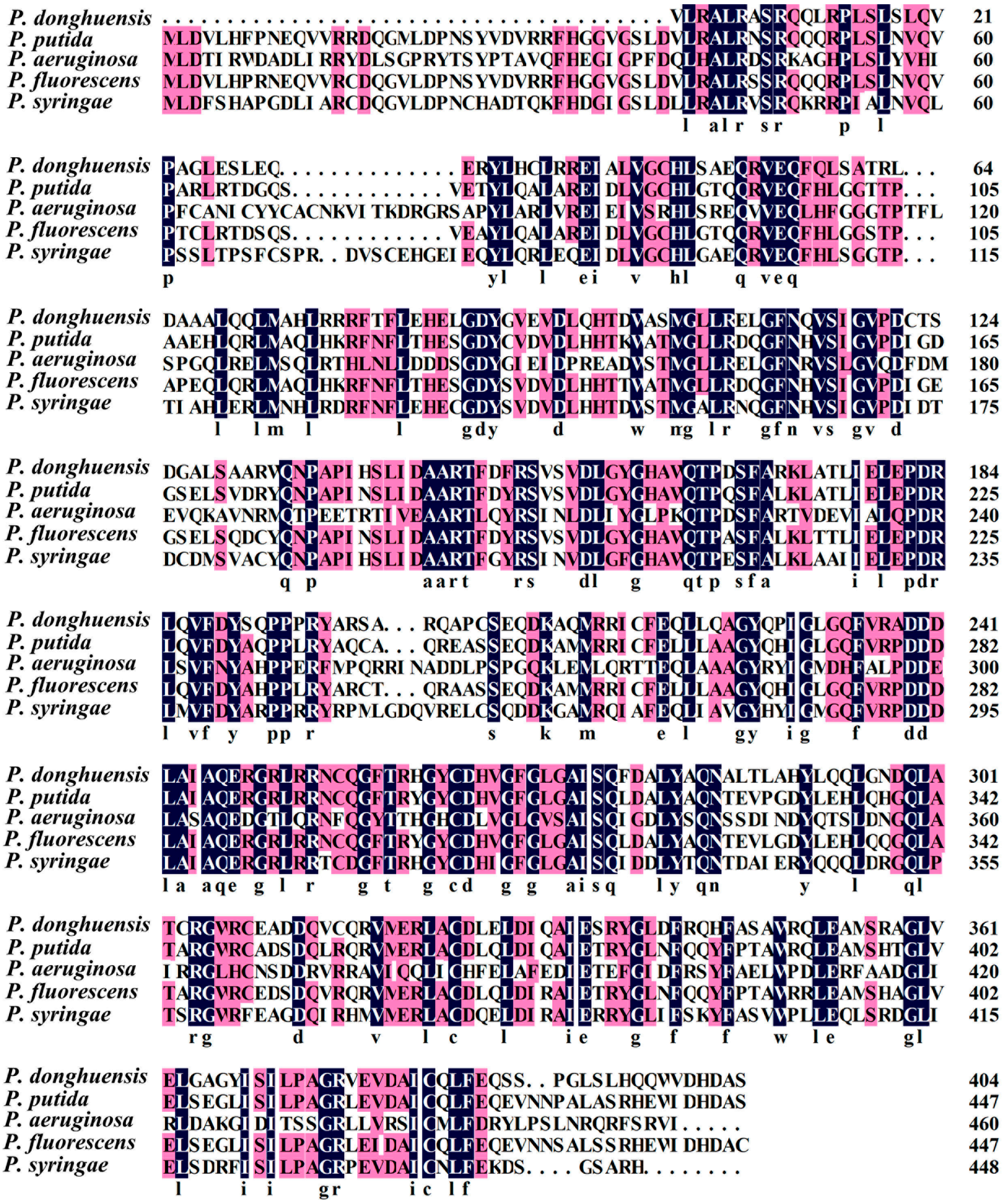

Figure S2. Sequence alignment of HemN2 proteins and highlight of the selected residues. Protein sequences for alignment are as follows: *P. donghuensis* HYS (WP_050990001.1), *P. putida* (WP_274294628.1), *P. aeruginosa* (RRJ56928.1), *P. fluorescens* (WP_191832076.1) and *P. syringae* (KNH28445.1).
